# Can the Systemic Immune-Inflammation Index Be a Prognostic Factor for Tympanoplasty Success?

**DOI:** 10.7759/cureus.91950

**Published:** 2025-09-10

**Authors:** Gökhan Toptaş, Mehmet Murat Günay, Melih Can Öner, Deniz Baklacı, Sibel Alicura Tokgöz, Ilker Akyıldız, Güleser Saylam

**Affiliations:** 1 Otolaryngology, Ankara Etlik City Hospital, Ankara, TUR; 2 Otolaryngology, Karaman Training and Research Hospital, Karaman, TUR; 3 Otolaryngology, Bülent Ecevit University Faculty of Medicine, Zonguldak, TUR; 4 Otolaryngology - Head and Neck Surgery, Lokman Hekim University, Faculty of Medicine, Ankara, TUR

**Keywords:** chronic otitis media, neutrophil-to-lymphocyte ratio (nlr), prognostic factors, systemic immune-inflammation index, tympanoplasty

## Abstract

Objective

To examine the role of the preoperative systemic immune-inflammation index (SII) in the success of type 1 tympanoplasty.

Methods

Between 2016 and 2022, 85 out of 442 patients who underwent type 1 tympanoplasty for chronic otitis media (COM) at our clinic met the inclusion criteria. Demographic data, preoperative complete blood count (CBC) results (including the neutrophil-to-lymphocyte ratio (NLR) and SII), audiological test results, computed tomography findings, and six-month follow-up results were evaluated.

Results

Anatomic outcomes were categorized into two groups based on graft success: Group 1 (64 patients with successful grafting) and Group 2 (21 patients with graft failure), yielding a success rate of 75.3%. Laboratory findings showed no significant differences in hemoglobin, platelet, or lymphocyte values (p>0.05), but neutrophil count (p < 0.05), NLR, and SII values (p < 0.05) were significantly higher in Group 2. The median SII was 509.1 (422.5-596.7) in Group 1 and 609.9 (521.8-790.5) in Group 2.

Conclusions

In our study, we found a negative correlation between SII, NLR, and type 1 tympanoplasty success. These parameters might be considered new prognostic inflammatory markers for tympanoplasty outcomes.

## Introduction

Chronic otitis media (COM) is a disease characterized by the persistent inflammation of the middle ear and mastoid cavity mucosa, lasting more than three months and accompanied by tympanic membrane perforation [1,2]. Particularly in developing countries, COM remains a significant health issue, and tympanoplasty is the primary treatment method [3-5]. The goal of tympanoplasty is to eliminate infection in the middle ear, restore any existing hearing loss, and provide a healthy middle ear cavity with an intact tympanic membrane [3,4,6]. While the anatomical success rate in tympanoplasty reaches 90% [6,7], factors influencing the success of this surgery, such as age, the size and location of the perforation, myringosclerosis, middle ear mucosa condition, contralateral ear status, and smoking, have been extensively studied in the literature [8-12].

Systemic inflammatory markers, including the neutrophil-to-lymphocyte ratio (NLR) and the platelet-to-lymphocyte ratio (PLR), provide important insights into an individual’s immune status. These inflammatory markers have been extensively studied for their prognostic value, especially in malignant tumors and diseases such as Bell’s palsy [13-15]. Another inflammatory index used for this purpose is the systemic immune-inflammation index (SII), defined as platelets x neutrophils / lymphocytes. SII has been investigated as a prognostic marker for inflammatory diseases and malignancies, showing a higher predictive value compared to NLR for many cancer types [15-18].

Despite previous research on the effect of the SII on diseases such as peripheral facial paralysis and sudden hearing loss, its role as a prognostic factor on graft success in tympanoplasty has not been explored [13,14,19].

The aim of this study was to evaluate the impact of preoperative SII and NLR values on graft success and hearing outcomes in patients undergoing type 1 tympanoplasty according to Wullstein classification.

## Materials and methods

Participants and study design

This study is a single-center, retrospective, observational investigation that complied with the tenets of the Declaration of Helsinki and received approval from our hospital. The requirement for written informed consent was waived due to the retrospective study design. The data of 442 patients who underwent tympanoplasty due to tubotympanic COM and who attended Ankara Diskapi Yildirim Beyazit Training and Research Hospital, a tertiary center, between 2016 and 2022, were analyzed, and 85 patients were included in the study. Preoperative complete blood count (CBC) results, audiological test results, computed tomography findings, operation notes, and postoperative sixth-month graft examination notes were retrospectively extracted from the medical records.

Based on their epicrisis notes, patients with tympanic membrane perforation lasting more than six months before surgery, no previous history of discharge, and preoperative audiological evaluation and thin-section temporal computed tomography imaging findings were identified. The demographic data of the patients, the location and size of the perforation, the status of the contralateral ear, the presence of myringosclerosis at admission, and the middle ear risk index (MERI) were evaluated [10]. The perforation of the tympanic membrane was assessed using a transcanal endoscopic examination. The tympanic membrane was divided into four quadrants according to the position of the malleus handle, and damage in any quadrant was defined as the perforation of the tympanic membrane. Patients with a MERI score of 1 (perforation: 1 point) were included in the study.

The patients’ preoperative CBC results, obtained the morning before surgery, were evaluated. CBC measurements were performed on an automatic hematology analyzer (CELL-DYN Ruby Hematology System, Illinois, USA), with analyses performed two hours after venous blood sampling. Hemoglobin, platelet, neutrophil, and lymphocyte (L) values were noted based on the CBC results. NLR and SII (calculated as platelets x neutrophils / lymphocytes) were derived from these values. Patients with hematological disorders, cardiac disease, renal insufficiency, autoimmune diseases, diabetes mellitus, hypertension, or a history of obstructive sleep apnea syndrome and those receiving steroid therapy were excluded from the study due to the potential effects of these conditions on hematological parameters.

Patients were excluded based on the following criteria: specific otologic pathologies, including chronic suppurative otitis media, cholesteatoma, complicated chronic otitis media, adhesive otitis media, retraction pockets, and marginal perforations; bilateral involvement or specific middle ear findings such as myringosclerosis or granulomatous/sclerotic mucosa; conductive comorbidities, namely sensorineural or mixed hearing loss, ossicular chain defects, or a history of otologic surgery; and significant nasal pathology, including severe septal deviation, turbinate hypertrophy, or polyps. Systemic exclusion criteria encompassed chronic medical conditions (e.g., diabetes mellitus, hypertension, cardiac disease), with the exception of those patients classified as ASA 1 by the attending anesthetist. Furthermore, active smokers and individuals lacking complete six-month postoperative follow-up data were also excluded.

Surgical technique and outcome measures

All surgeries were performed at our clinic under general anesthesia by two otolaryngologists, each possessing over five years of experience and comparable surgical expertise. The procedure involved a microscopic approach via a postauricular incision. Temporal fascia was employed as the graft material. After perforation debridement and elevation of the tympanomeatal flap, all patients were evaluated for middle ear mucosa and ossicular chain integrity. Ossicular chain pathologies were excluded by observing round window movement through palpation of the malleus. Subsequently, the temporalis fascia graft was carefully positioned using the over-underlay technique. In this approach, the graft was placed lateral to the handle of the malleus to ensure adequate contact and stability, while simultaneously being positioned medial to the tympanic annulus to provide proper integration with the native tympanic membrane remnant. This dual-plane placement was intended to maximize graft adherence, minimize the risk of medialization or lateralization, and optimize the functional outcome of tympanic membrane reconstruction.

The patients were evaluated at the postoperative first week, first month, third month, and sixth month. At the sixth-month follow-up, cases presenting with an intact graft in proper position and a well-aerated middle ear were considered surgically successful. In addition, the patients’ hearing was assessed at the sixth-month follow-up using pure-tone audiometry (PTA). PTA values were calculated as the average thresholds at four frequencies (500, 1,000, 2,000, and 4,000 Hz). The preoperative and postoperative air conduction thresholds, the mean air-bone gap, and the mean air-bone gap gain were determined. Hearing success was defined as a PTA air-bone gap of 10 dB or less at the sixth-month follow-up visit.

The effect of SII, NLR, and lymphocyte, neutrophil, and platelet counts on the anatomical and functional outcomes of tympanoplasty was evaluated.

Statistical analysis

The analysis of the results was performed using the IBM SPSS Statistics version 21.0 software for Windows (IBM Corp., Armonk, NY). The data were tested for a normal distribution using the Kolmogorov-Smirnov test. To investigate the differences between groups, the Mann-Whitney U test was used for two groups and the Wilcoxon test for related samples. The chi-square test was performed for categorical variables. Statistical significance was defined as p < 0.05.

## Results

A total of 85 patients were included in the study. Of these, 58 (68.2%) were female with a mean age of 35.5 ± 12.4 years (range: 18-61), and 27 (31.8%) were male with a mean age of 33.8 ± 12.9 years (range: 18-58). Tympanic membrane perforation was located in the right ear in 49 patients (57.6%) and in the left ear in 36 patients (42.4%). The preoperative perforation sizes and locations are shown in Table 1.

**Table 1:** Preoperative perforation sizes and locations of the study groups Group 1: intact graft; Group 2: perforated graft *Chi-square / Fisher exact test

Parameters		Group 1; n = 64 (75.3%)	Group 2; n = 21 (24.7%)	Chi-square value	P value*
Preoperative perforation size	<50%	31 (48.4%)	7 (33%)	1.487	0.48
	50-75%	22 (34.4%)	9 (42.9%)		
	>75%	11 (17.2%)	5 (23.8%)		
Preoperative location of perforation	Anteroinferior	23 (35.9%)	6 (28.6%)	4.344	0.12
	Posteroinferior	20 (31.3%)	3 (14.3%)		
	Subtotal	21 (32.8%)	12 (57.1%)		

The anatomical results were divided into two groups based on graft success. Group 1 comprised 64 patients with successful grafting, while the remaining 21 patients were classified as Group 2. The graft success rate was determined to be 75.3%. There was no significant difference between the intact graft group (Group 1) and the perforated graft group (Group 2) in terms of gender or age (p = 0.72 and 0.07, respectively).

Upon examining laboratory findings, there were no statistically significant differences in hemoglobin, platelet, and lymphocyte values between the two groups (p > 0.05). However, the neutrophil count (p < 0.05), NLR, and SII values (p < 0.05) were significantly higher in Group 2. The median SII value was 509.1 (422.5-596.7) in Group 1 and 609.9 (521.8-790.5) in Group 2. The laboratory findings are summarized in Table 2.

**Table 2:** Hematological parameters of the study groups Variables were presented as median (IQR). Group 1: intact graft; Group 2: perforated graft; SD: standard deviation; NLR: neutrophil-to-lymphocyte ratio; SII: systemic immune-inflammation index **Mann-Whitney U test

Variables	Group 1 (n=64)	Group 2 (n=21)	U value	P value**
Hemoglobin	13.5 (12.7-15.7)	13.6 (12.5-15.4)	637.5	0.804
Platelet count (10^3^/μL)	241.0 (199.3-301.5)	255.0 (227.5-349.5)	514.0	0.107
Neutrophil count (10^3^/μL)	4.5 (3.6-5.2)	5.5 (4.2-6.4)	421.0	0.011
Lymphocyte count (10^3^/μL)	2.0 (1.7-2.6)	2.3 (1.8-3.3)	539.0	0.175
NLR	1.9 (1.5-2.3)	2.4 (1.7-3.3)	424.0	0.012
SII	509.1 (422.5-596.7)	609.9 (521.8-790.5)	336.0	0.001

Hearing outcomes

There were no significant differences between the two groups in terms of the preoperative air and bone PTA values (p = 0.22 and 0.11, respectively). In Group 1, the mean preoperative air PTA value was 36.17 ± 10.9, and the mean postoperative air PTA value was 19.5 ± 7.6 (p < 0.01). In the same group, the mean preoperative and postoperative bone PTA values were 12.6 ± 6.3 and 12.1 ± 6.1, respectively (p = 0.27), and the mean air-bone gap improvement was 16.6 ± 6.7. In Group 2, the mean preoperative and postoperative air PTA values were 32.7 ± 8.6 and 31.5 ± 8.4, respectively (p = 0.8), and the mean preoperative and postoperative bone PTA values were 9.9 ± 5.4 and 9.8 ± 5.3, respectively (p = 0.32), indicating no air-bone gap improvement. Hearing outcomes are presented in Tables 3-4.

**Table 3:** Air PTA outcomes of study groups Group 1: intact graft; Group 2: perforated graft; SD: standard deviation; PTA: pure-tone average *** Wilcoxon test

	Preoperative Air PTA (dB) (mean ± SD)	Postoperative Air PTA (dB) (mean ± SD)	W value	P value (Pre-Post)***
Group 1	36.17 ± 10.9	19.5 ± 7.6	1.0	<0.01
Group 2	32.7 ± 8.6	31.5 ± 8.4	0.06	0.82

**Table 4:** Bone PTA outcomes of study groups Group 1: intact graft; Group 2: perforated graft; SD: standard deviation; PTA: pure-tone average. *** Wilcoxon test

	Preoperative Bone PTA (dB) (mean ± SD)	Postoperative Bone PTA (dB) (mean ± SD)	W value	P value (Pre-Post)***
Group 1	12.6 ± 6.3	12.1 ± 6.1	0.33	0.27
Group 2	9.9 ± 5.4	9.8 ± 5.3	0.11	0.32

## Discussion

In our study, we investigated the effect of hematological biomarkers, namely NLR and SII, on the success rate of type 1 tympanoplasty and found that they were negatively correlated with the success of this surgery. There were no significant differences between the intact and perforated graft groups in relation to hemoglobin, platelet, and lymphocyte values. It seems that SII, a new and cost-effective marker that can be rapidly calculated, could be used as a new prognostic factor for tympanoplasty success.

Determining factors that may influence surgical success before tympanoplasty is crucial for surgical planning. In the literature, graft success rates vary considerably (ranging from 50% to 99%) [4-12]. Factors such as the location and size of tympanic membrane perforation, the presence of myringosclerosis in the residual membrane, mucosal disease in the middle ear, otorrhea, the characteristics of the graft material used, the presence of pathology in the contralateral ear, surgical experience, and preference for endoscopic or microscopic surgical methods have been extensively studied [4-13]. Patients with parameters known to affect graft success and mentioned above (excluding perforation size and location) were excluded from our study to assess the impact of SII and NLR on graft success in a more homogeneous group. However, our study did not find a statistically significant correlation between perforation size or location and graft success rate.

In tympanoplasty, factors influencing surgical success have often been studied in terms of disease prevalence and the efficacy of surgical methods. However, host-related factors, immunity, and inflammation also play crucial roles in wound healing. The goal of tympanoplasty is to achieve epithelialization using graft material to create an intact barrier between the external auditory canal and the middle ear cavity. Immediately after surgery, inflammation in the surgical area activates platelets, promoting clotting and attracting neutrophils and monocytes to migrate to the surgical site. This early inflammatory phase transitions to granulation tissue formation, where fibroblasts produce collagen fibers that contribute to wound contraction and tensile strength. In this phase, regeneration begins at the edges of the wound, and epithelialization progresses over the formed granulation tissue. The migration of epithelial cells continues until they converge. Once granulation tissue forms and epithelialization is complete, the proliferation phase ends. Following the proliferation phase, remodeling and maturation commence, which may last for years. However, the wound typically regains around 95% of its initial strength after approximately six weeks [20,21].

The host’s immunity plays a significantly influential role in the aforementioned inflammatory process. Numerous studies have focused on determining the host’s immune response. Systemic inflammatory markers, such as neutrophil count, platelet count, lymphocyte count, NLR, PLR, and particularly, SII, which has gained prominence in recent years, provide important insights into an individual’s immune status. The immune response, triggered against various components, can either protect the organism or lead to harmful results. High NLR and SII values have been shown to be associated with vascular, thromboembolic, and ischemic diseases. An increase in neutrophils leads to an elevation in reactive oxygen radicals, triggering inflammation [22,23].

Several studies suggest that the cell-mediated inflammatory process may be a key factor in the pathogenesis of Bell’s palsy and sudden hearing loss. Bucak et al. and Baklacı et al. found higher NLR values in patients with Bell’s palsy and noted a statistically significant negative correlation between insufficient recovery and these elevated NLR levels [13,24]. On the other hand, research on malignancies has indicated that elevated SII values are associated with a poor prognosis [15-18,25]. 

Although not specifically in the otolaryngology literature, there are studies correlating high SII levels with surgical failure. Somay et al. demonstrated that high SII values decreased the success rates of temporomandibular joint arthrocentesis [26]. Additionally, Erdogan et al. reported reduced success in varicocelectomy procedures, while Özsoy et al. found lower surgical success rates in cases of internal urethrotomy with high SII values [27,28]. Feng et al. recorded an increase in postoperative infectious complications in colorectal cancer cases with high preoperative SII values [29]. These findings can be attributed to the increase in systemic inflammation and local inflammation in the surgical field, leading to an increase in microthrombosis with high platelet levels, an increase in free oxygen radicals with high neutrophil levels, as well as the inadequate inhibition of harmful inflammation with low lymphocyte levels. We consider that the high SII values in our study likely reduced the success rate of type 1 tympanoplasty surgery for similar reasons.

The absence of a prospective design had a particular impact on the comparability of postoperative CBC results. Therefore, further prospective studies are needed to better understand the role of SII in tympanoplasty success.

Limitations

The present study has some limitations. Its retrospective, single-center design and relatively small sample size may restrict the generalizability of the findings.

## Conclusions

To our knowledge, this study is the first to investigate the association between the hematological biomarkers SII and NLR and the success of tympanoplasty. High SII and NLR values were determined to have a negative correlation with the intact graft rate. These parameters might be considered new prognostic inflammatory markers indicating the success of tympanoplasty. Further prospective studies are needed to better understand the role of SII in surgical healing.
